# Next-generation in vitro blood–brain barrier models: benchmarking and improving model accuracy

**DOI:** 10.1186/s12987-021-00291-y

**Published:** 2021-12-07

**Authors:** Raleigh M. Linville, Peter C. Searson

**Affiliations:** 1 Institute for Nanobiotechnology, Johns Hopkins University, Baltimore, MD USA; 2 Department of Biomedical Engineering, Johns Hopkins University, Baltimore, MD USA; 3 Department of Materials Science and Engineering, Johns Hopkins University, Baltimore, MD USA

**Keywords:** Blood–brain barrier, Brain microvascular endothelial cells, Induced pluripotent stem cells, Microenvironment, Microenvironmental cues, Model accuracy, Differentiation, Gene expression

## Abstract

With the limitations associated with post-mortem tissue and animal models, In vitro BBB models enable precise control of independent variables and microenvironmental cues, and hence play an important role in studying the BBB. Advances in stem cell technology and tissue engineering provide the tools to create next-generation in vitro BBB models with spatial organization of different cell types in 3D microenvironments that more closely match the human brain. These models will be capable of assessing the physiological and pathological responses to different perturbations relevant to health and disease. Here, we review the factors that determine the accuracy of in vitro BBB models, and describe how these factors will guide the development of next-generation models. Improving the accuracy of cell sources and microenvironmental cues will enable in vitro BBB models with improved accuracy and specificity to study processes and phenomena associated with zonation, brain region, age, sex, ethnicity, and disease state.

## Background

Establishing how the blood–brain barrier (BBB) responds to different chemical, physical, and biological perturbations is key to understanding the link between blood–brain barrier (BBB) health and brain health. In vitro BBB models enable independent control over the spatial organization of cellular components and microenvironmental cues, and hence are complementary to animal models, which display species-dependent differences, and studies in humans where imaging has limited resolution and tissue samples are difficult to obtain [1]. Highly reductive in vitro models formed by confluent monolayers of brain microvascular endothelial cells (BMECs) on transwells enable high-throughput screening of specific functional properties, however, more complex models are needed to study a broader range of biological processes and to recapitulate the physiological and pathological responses of human brain endothelium. Recently, advances in stem cell technology and tissue engineering have provided the tools to create these models. Here, we consider the important factors in establishing the accuracy of BBB models, with a focus on tissue-engineered models incorporating stem-cell derived BMEC-like cells (iBMECs). The emerging understanding of the human BBB from single-cell/nucleus sequencing studies provides the foundations to benchmark in vitro models with human, zonation, brain region, ethnicity, sex, age, and disease state specificity. Key factors in developing these next-generation models include: (1) improved characterization of the human BBB for accurate benchmarking of in vitro models, (2) improved cell engineering techniques to mimic gene and protein expression of BMECs and supporting cell types, and (3) improved tissue engineering methods to mimic microenvironmental cues present within the cerebrovasculature.

## Accuracy of in vitro BBB models

### Defining accuracy

The observable characteristics of an in vitro BBB model are derived from two factors: the cellular components (e.g., protein and gene expression profiles) and the local microenvironment established in the model. These two factors are interrelated since the microenvironment can have significant influence on the protein and gene expression profiles of the source cells. The observable characteristics of the model encompass the functional responses to physiological, therapeutic, or pathological perturbations (e.g., barrier function, response to cytokines, stress response, etc.) Therefore, the accuracy of a BBB model can be defined as the level to which the observable characteristics recapitulate the human BBB. In principle, the accuracy can be quantified with values expected to be between 0 and 1. However, establishing the accuracy of in vitro BBB models has been challenging as the observable characteristics of the human BBB are limited and/or inconsistent. For example, the permeability of small molecular weight compounds is inconsistently reported in animal models [2, 3], and has not been directly measured in humans. To overcome this limitation, comparison of the gene expression profiles of endothelial cells in vitro and in the human brain (transcriptomic accuracy) has emerged as a useful proxy for model accuracy [4, 5]. With the advent of single cell and single nucleus RNA sequencing methods, gene expression of cell types in the BBB can be assessed from human brain tissue. However, the relationship between gene expression and protein expression depends on many factors including post-transcriptional regulation, protein secretion, and RNA/protein degradation [6], and hence transcriptomic accuracy should be assessed in the context of protein expression and functional responses.

### How accurate do BBB models need to be?

The transwell model, incorporating a monolayer of Madin Derby Canine Kidney epithelial cells (MDCKs, established in 1958), has been a workhorse in BBB research for many years. This model is widely used for measurement of solute permeability, assessment of transendothelial cell migration, as well as for many other biologically- and clinically-relevant processes. Although MDCKs are epithelial cells, they express tight junctions and hence paracellular transport across confluent monolayers is negligible. As a consequence, solute transport is dominated by active transport or passive diffusion across the apical and basolateral cell membranes [7]. Assuming that differences in lipid composition do not influence kinetics, passive transport across MDCK monolayers is considered to mimic passive transport across endothelial monolayers in the brain. MDCK cells have also been genetically engineered to assess whether small molecule solutes are substrates for P-gp efflux pump. Primary and immortalized brain microvascular endothelial cells (BMECs) have also been widely used in BBB models but often show batch-to-batch variability, loss of barrier function during ex vivo culture, and very low transendothelial electrical resistance (TEER), a measure of paracellular barrier integrity [8, 9]. Despite these limitations, these cell sources have enabled important foundational studies of the BBB, especially related to solute transport and cell transmigration.

From an engineering perspective, in vitro models only need to recapitulate the particular function of interest. For example, transwell models, while highly reductive, enable rapid assessment of solute permeability across endothelial and epithelial barriers. Similarly, microfluidic organ-on-a-chip devices (e.g., lung-on-a-chip, liver-on-a-chip, etc.) do not attempt to recapitulate all aspects of organ function but are invaluable for high-throughput screening of specific processes or functions, or in answering specific research questions. Due to the complexity of the human BBB, in vitro models should be engineered to answer specific research questions, acknowledging that over-engineering is wasted effort and that under-engineering may compromise the relevance of findings. Establishing the accuracy of BBB models is a major challenge and will require protocols for assessing the response to different perturbations or stresses, e.g., physical forces (e.g., mechanical forces, electromagnetic radiation, temperature, hypoxia), endogenous factors (e.g., associated with lifestyle or disease), exogenous factors (e.g., therapeutics), chemical factors (e.g., ROS, inflammatory factors), or toxins and pathogens.

### The challenges in establishing model accuracy

As described above, the accuracy of an in vitro BBB model is dependent on the cellular components and the local microenvironment, which together define the observable characteristics. Along the arterio-venous (AV) axis, from arterioles to capillaries to venules, there are significant differences in perivascular and mural cell organization, blood flow patterns, and association with neurons and glial cells [10]. In pre-capillary arterioles, the endothelium is surrounded by smooth muscle cells (SMCs) among other supporting cell types, and experiences a relatively high shear stress. In capillaries, BMECs wrap around to form tight junctions with themselves and their upstream and downstream neighbors, and are surrounded by pericytes and astrocyte end-feet. In venules, BMECs are directly surrounded by mural cells distinct from their arteriolar neighbors and a perivascular space located between the basement membrane and astrocytic glia limitans. Some biological functions occur preferentially at different locations along the AV axis, including trafficking of immune cells, cancer cells, pathogens, plasma, and nanoparticles [11–13]. Many other aspects of the microenvironment, including physical dimensions, flow rates, pressure, shear stress, and neurovascular coupling [14] vary along the AV axis and provide important inputs for the engineering design of BBB models.

Recent single cell transcriptomic studies have established that gene expression of BMECs in the brain varies along the AV axis both in mice [13, 15, 16] and humans [4, 5]. Species-dependent differences between BMECs in the mouse and human brain have been reported to include  ~ 10% of all genes [4], but the implications of these differences remain to be fully explored. Along the AV axis, BMECs and mural cells display zonated gene and protein expression which underlie distinct biological functions, as described above [4, 5, 13, 15, 16]. However, these studies are based on relatively small human sample sizes and different isolation protocols, which makes direct comparison challenging. In addition, limited access to non-pathological human brain tissue, also contributes to the difficulty in benchmarking BMECs in models to BMECs in the human brain. Ultimately, there is a need to establish datasets from multiple individuals using standardized protocols. In addition to zonation specificity, the observed characteristics of the human BBB are dependent on many factors, including brain region, age, sex, ethnicity, and disease state. In particular, differences associated with sex and ethnicity are not yet well understood, and hence benchmarking is currently not possible. However, in vitro models incorporating iPSCs across these variables could contribute to our understanding and guide future animal and human studies.

In summary, next-generation in vitro models of the BBB should consider zonation, brain region, age, sex, ethnicity, and disease state specificity. While variations associated with these factors demonstrate that it is not possible to design a universal BBB model, for some applications generic or reductive models will be more appropriate. For example, a generic model may recapitulate BMEC barrier integrity at the level of capillaries and be sufficient for studies of drug delivery. Similarly, a reductive model may incorporate multiple cell types but in a non-physiological geometry (e.g., 2D). Next, we review two major contributors to model accuracy: cell source and microenvironment.

## Accuracy of the cell source

To overcome limitations associated with MDCKs and primary/immortalized cell lines, and to provide a reproducible and scalable source of cells for BBB research, various differentiation strategies have been developed to generate BMEC-like cells (iBMECs) from induced pluripotent or embryonic stem cells [17]. Many studies have established that confluent monolayers of iBMECs display expression of important EC- and BBB-specific markers at the protein and gene level [17]. To date, the observable characteristics of BBB models using iBMECs have been based on a limited set of functional assays, primarily related to barrier integrity, that recapitulate observations in animal models and/or humans, including high TEER, low solute permeability, and efflux activity [11]. The validation of aspects of BBB function has enabled new paradigm for BBB research where pathological mechanisms are first studied in tissue-engineered iBMEC-based models, taking advantage of the ability to have independent control over experimental variables, and then subsequently verified in animal models or human tissue [18–20].

However, recent comparisons to primary BMECs and other endothelial cell types, show that iBMECs differentiated using current protocols possess reduced endothelial identity (e.g., lower gene and protein expression of VE-cadherin compared to other endothelial sources) [17] and elements of epithelial identity (e.g., gene and protein expression of E-cadherin) [21]. Furthermore, the gene expression profiles for iBMECs have not yet been benchmarked to human single-cell BMEC datasets, a key step is establishing model accuracy. Thus, driving iBMEC gene expression towards that of human BMECs and understanding the heterogeneity of differentiated cells will be key towards improving the accuracy of in vitro BBB models.

**Fig. 1:**
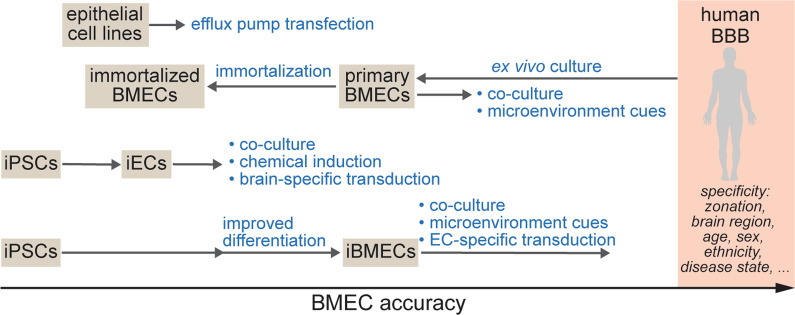
Trajectories for generating cells for accurate BMECs for in vitro BBB models. The relative position of cell sources is dependent on the specific observable characteristics, while the target comparison to human BMECs is specific to zonation, brain region, age, sex, ethnicity, and disease state. Cell engineering approaches can improve aspects of BBB model accuracy while also impairing other aspects. Achieving high accuracy will require further developments in cell and tissue engineering

There are several strategies for driving the identity of iBMECs further towards human BMECs, including (Fig. 1): (1) transcription factor (TF) reprogramming, (2) chemical induction, and (3) the development of novel differentiation approaches that better mimic BBB development [21–26]. Each of these approaches has had success in enhancing BMEC identity, although high levels of similarity to human BMECs has not yet been achieved. TF reprogramming has been used to overexpress brain-specific transcription factors (e.g., *SOX18* and *TAL1*) in non-brain specific iECs which transiently increases TEER and gene expression of BBB markers [22]. iBMECs can be reprogrammed with ETS TFs (e.g., *ETV2*, *ERG*, *FLI1*) to improve endothelial identity, however this process is associated with a loss of barrier function and leads to gene expression profiles more closely resembling non-brain specific iECs [21, 27]. TGF-β inhibition can enhance endothelial and BBB-specificity of iECs and iBMECs [24, 25], and activation of Wnt/β-catenin signaling (using agonist of Wnt/β-catenin signaling, Wnt ligands, or conditioned media from neural progenitor cells) in endothelial progenitor cells upregulates BBB-specific gene expression [26]. While these approaches hold promise and have shown improvements in accuracy of specific BBB or endothelial genes and/or functional properties, they have so far been unable to achieve the physiological barrier properties typical of iBMEC monolayers, and show TEER values comparable to primary and immortalized BMEC sources.

Benchmarking iBMECs to human BMECs will be increasingly challenging due to the emerging understanding of the functional differences associated with zonation, brain region, age, and other factors, as well as the variation between individuals within each group. Therefore, the transcriptomic accuracy of iBMECs will depend on the particular research question to be addressed. For example, recent sequencing data enables benchmarking iBMECs to achieve zonation-specific identity [4, 5]. The relationship between transcriptomic accuracy of source cells and model accuracy will also be important in creating next-generation models. While increasing transcriptomic accuracy will, in most cases, lead to increased accuracy of the BBB model, it will also depend on the specific genes that are congruent with the target BMECs and hence the biological processes that are recapitulated. Finally, this complexity also makes it difficult to establish the transcriptomic identity of “generic” iBMECs that can be used to study processes where differences associated with zonation, for example, are not important.

## Accuracy of the microenvironment

The local microenvironment plays an important role in defining BBB model accuracy. When primary BMECs are cultured ex vivo they lose BBB-specific transcripts, while endothelial transcripts are less significantly altered [9]. These changes demonstrate the critical importance of microenvironmental cues in maintenance of BBB identity. However, recapitulating the cerebrovascular microenvironment poses many challenges, as outlined below. The brain is characterized by a very high density of neurons and glial cells with an extracellular space (~ 20 volume %) filled with interstitial fluid containing long-chain macromolecules, primarily proteoglycans. However, the brain does not have a bulk structural extracellular matrix (ECM) as in other tissues. Therefore, to provide structural support for cell culture, in vitro BBB models have largely employed an extracellular matrix material (e.g., collagen, fibrin, etc.) that matches the stiffness of brain tissue. Approaches based on spheroids, which eliminates the need for a structural matrix, are better able to recapitulate the cellular organization in the brain, but vascularization and perfusion of the spheroid core remain a significant challenge.

**Fig. 2:**
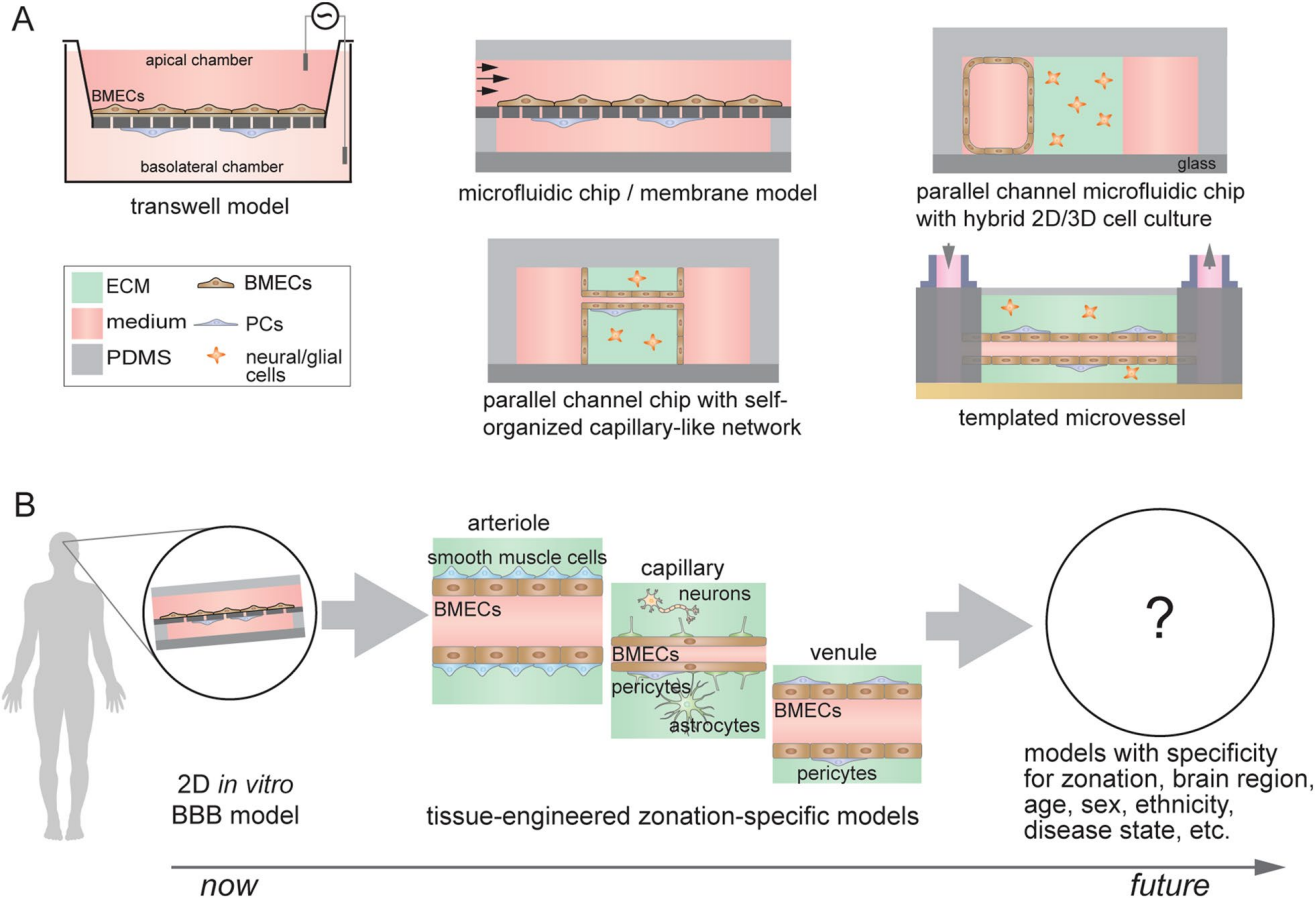
Current and future in vitro BBB models. **A** Examples of in vitro BBB models: transwell model, microfluidic chip/membrane model, microfluidic parallel channel models, and templated model. These models utilize different engineering strategies to mimic selected aspects of the cerebrovascular microenvironment. **B** Towards next-generation models: to recapitulate specific biological processes occurring along the arterio-venous axis and with specificity for brain region, age, sex, ethnicity, and disease state will require more complex models that recapitulate the local microenvironment, including the spatial organization of cells, flow, blood components, etc.

In addition to the zonation specificity of cell types and their spatial arrangement, other important microenvironmental cues that regulate BMEC phenotype include basement membrane [28], shear stress [29, 30], interactions with other cell types (e.g., mural cells) [31, 32], cylindrical geometry [33], blood composition [34, 35], and neuronal activity [36]. Tissue-engineered in vitro models of the BBB provide an opportunity to explore the role of microenvironment on BBB model accuracy in detail, with the key advantage of independent control of experimental variables to assess individual and synergistic effects on observable characteristics. Current in vitro model designs capture different aspects of these microenvironmental cues (Fig. 2A) [37], and can be broadly categorized as: (1) 2D microfluidic chip membrane-based models that incorporate shear flow and other cell types [30, 38], (2) parallel channel microfluidic models will hybrid 2D/3D cell culture [39, 40], (3) parallel channel microfluidic models with self-organized microvascular networks [20, 31], and (4) templating based devices which generate cylindrical microvessels in an extracellular matrix [41, 42]. Self-organization and templating approaches enable incorporation of a wide repertoire of microenvironmental cues including cylindrical geometry, cell-ECM interactions, and direct cell–cell interactions. These cues can enhance BBB model accuracy compared to shear stress alone, including enriched endothelial identity, unique cytokine and angiogenic responses, and lower paracellular permeability [27]. Across model types, co-cultured pericytes and astrocytes enhance expression of BBB markers and increase barrier tightness when source BMECs have poor barrier function (e.g., low TEER) [31, 42], while these same effects are attenuated when high TEER is achieved at baseline [11, 32].

Despite advances in tissue engineering, BBB models do not fully recapitulate the microenvironment of the BBB in the human brain. Some important challenges include (Fig. 2B): (1) improved fidelity of the spatial arrangement of BBB cell types in a zonation-specific manner, (2) incorporation of blood components known to be important in communication between the vascular system and the brain, (3) developing strategies for multiscale/hierarchical models along cerebrovascular zones, and (4) recapitulating aspects of neurovascular coupling. While advances in tissue engineering support the feasibility of addressing these challenges, success will require a significant effort within the BBB research community.

## Conclusions

Creating next-generation in vitro BBB models will require integration of knowledge from vascular atlases of protein and gene expression in the human brain and the emerging understanding of the role of a broad range of extrinsic factors (microenvironmental cues, supporting cell types, blood components, etc.) on observable characteristics. Stem cell technology provides a pathway for engineering iBMECs and isogenic supporting cells with high transcriptomic similarity to cells in the human brain, but will require advances in cell engineering. Advances in biomaterials and tissue engineering have improved the toolkit for incorporating microenvironmental cues into BBB models, however, significant advances are needed to further improve model accuracy. Despite these challenges, next-generation BBB models will enable studies of diseases of the brain, responses to a wide range of physical, chemical, and biological perturbations, and delivery of therapeutics to the brain. Next-generation models will improve accuracy of the gene expression of cell sources and microenvironments to engineer models that achieve high human-specificity, zonation-specificity, brain region-specificity, and disease state-specificity.

## Data Availability

Not applicable.
